# ISG15 predicts poor prognosis and promotes cancer stem cell phenotype in nasopharyngeal carcinoma

**DOI:** 10.18632/oncotarget.7626

**Published:** 2016-02-23

**Authors:** Ren-Hui Chen, Yong Du, Ping Han, Hong-Bo Wang, Fa-Ya Liang, Guo-Kai Feng, Ai-Jun Zhou, Mu-Yan Cai, Qian Zhong, Mu-Sheng Zeng, Xiao-Ming Huang

**Affiliations:** ^1^ Department of Otolaryngology-Head and Neck Surgery, Guangdong Provincial Key Laboratory of Malignant Tumor Epigenetics and Gene Regulation, Medical Research Center, Sun Yat-sen Memorial Hospital, Sun Yat-sen University, Guangzhou, 510120, China; ^2^ Department of Experimental Research, State Key Laboratory of Oncology in South China, Collaborative Innovation Center for Cancer Medicine, Sun Yat-sen University Cancer Center, Guangzhou 510060, China; ^3^ Department of Pathology, State Key Laboratory of Oncology in South China, Collaborative Innovation Center for Cancer Medicine, Sun Yat-sen University Cancer Center, Guangzhou 510060, China

**Keywords:** interferon-stimulated gene 15, nasopharyngeal carcinoma, prognosis, cancer stem cell

## Abstract

Interferon-stimulated gene 15 (ISG15), the first identified ubiquitin-like protein, is known for its anti-viral capacity. However, its role in tumorigenesis remains controversial. Here, using RNA-seq profiling analysis, we identified ISG15 as a differentially expressed gene in nasopharyngeal carcinoma (NPC) and validated its overexpression in NPC samples and cells. High ISG15 levels in NPC tissues were correlated with more frequent local recurrence and shorter overall survival and disease-free survival. ISG15 overexpression promoted a cancer stem cell phenotype in NPC cells, including increased colony and tumorsphere formation abilities, pluripotency-associated genes expression, and *in vivo* tumorigenicity. By contrast, knockdown of ISG15 attenuated stemness characteristics in NPC cells. Furthermore, overexpression of ISG15 increased NPC cell resistance to radiation and cisplatin (DDP) treatment. Our study demonstrates a protumor role of ISG15, and suggests that ISG15 is a prognostic predictor and a potential therapeutic target for NPC.

## INTRODUCTION

Nasopharyngeal carcinoma (NPC) is a prevalent disease in Southern China and Southeast Asia [1]. Despite the improved survival of NPC patients with advances in treatment techniques, such as radiotherapy and a combination of chemotherapy, the prognosis of advanced stages is less favorable [2, 3]. Locoregional recurrence and distant metastases remain the major etiology of mortality in NPC patients [4–6]. Therefore, identifying diagnostic markers and therapeutic targets for NPC is a novel management strategy in the era of genomics. Using RNA-seq profiling analysis, we identified Interferon Stimulated Gene 15 (ISG15) as one of the differentially expressed genes in NPC. ISG15 is the first identified ubiquitin-like protein that is conjugated to cellular substrates to form ISGylated proteins and shows antiviral and anti-bacterial activities [7–11]. Recent studies have reported that ISG15 is frequently overexpressed in various cancers [12–15]. Unlike its definite anti-viral capacity, the role of ISG15 in tumor development remains controversial. In cancer cells, ISG15 exists in two forms: free (intracellular) and conjugated to cellular proteins (ISGylation) [16]. Intracellular ISG15 exhibits protumor capacities such as promotion of the proliferation and migration of cancer cells [12, 14, 15, 17]. However, it has also been reported that ISGytion attenuates the HIF-1a mediated tumorigenic growth [18] and suppresses lung cancer growth by targeting cyclin D1 [19]. In addition to the intracellular system, free ISG15 is also secreted into the extracellular milieu and acts as an immunomodulatory cytokine in the tumor microenvironment [13, 20]. It has been reported that tumor-associated macrophages (TAMs) secrete ISG15, which enhances cancer stem cell (CSC) phenotypes in pancreatic ductal adenocarcinoma [13]. While Burks and colleagues have demonstrated extracellular free ISG15 triggers an antitumor immune response and suppresses breast cancer growth [20]. Therefore, whether ISG15 is a tumor promoter or a tumor suppressor has not yet been fully established.

In the present study, we showed that ISG15 was markedly overexpressed in NPC. Increased ISG15 expression was correlated with tumor recurrence and poor prognosis, which could be due to its capacity to promote CSC phenotype and radioresistance and chemoresistance in NPC. Our findings provide new insight into the role of ISG15 in NPC tumorigenesis and suggest that ISG15 might be a potential therapeutic target for NPC.

## RESULTS

### ISG15 was identified as a potential differentially expressed gene in NPC through RNA-Seq

**Figure 1:**
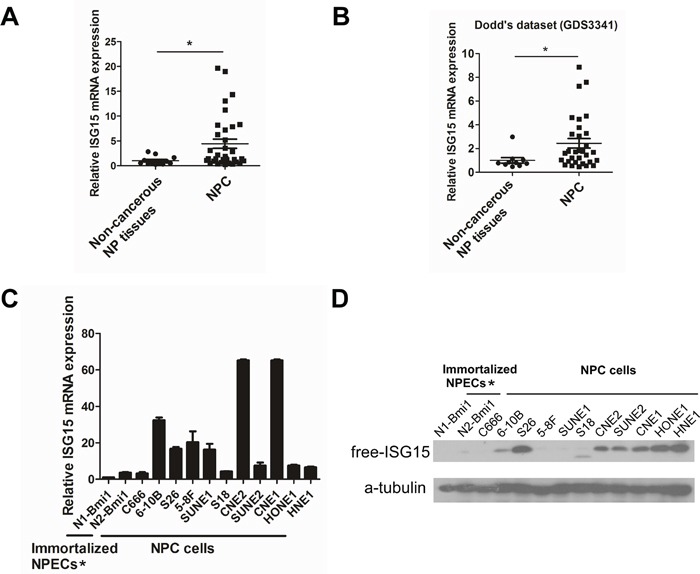
Up-regulation of ISG15 expression in NPC sample and cell lines **A.** Expression of ISG15 mRNA was detected by RT-PCR in NPC and NP mucosa samples. **B.** Expression of ISG15 mRNA from Dodd's dataset for NPC versus NP mucosa samples. **C.** ISG15 mRNA expression and **D.** protein level in NPC cell lines and immortalized NPECs. * Immortalized NPECs, NPEC1-Bmi1 and NPEC2-Bmi1, have been abbreviated as N1-Bmi1 and N2-Bmi1.

To investigate genes capable of conferring tumorigenesis in NPC, we selected a panel of nine patients' nasopharyngeal biopsy specimens and one primary cultured nasopharyngeal epithelial cell (NPEC03) for RNA-Seq analysis. ISG15 was identified as one of the differentially expressed genes in NPC. To validate the RNA-Seq data, qRT-PCR and Western blotting were performed to detect ISG15 expression in two cohorts of NPC biopsy specimens and non-cancerous nasopharyngeal epithelium specimens, and NPC cell lines as well as immortalized nasopharyngeal epithelial cell lines (NPECs). We demonstrated that ISG15 mRNA was significantly up-regulated in tumor tissues (Figure 1A). These results were consistent with Dodd's outcome from the publically available microarray GEO dataset (Figure 1B, http://www.ncbi.nlm.nih.gov/sites/GDSbrowser?acc=GDS3341). In addition, ISG15 mRNA and protein levels were elevated in multiple NPC cell lines, such as SUNE2, 6-10B, S26, CNE1, CNE2, HNE1 and HONE1, compared with immortalized NPECs, NPEC1-Bmi1 and NPEC2-Bmi1 (Figure 1C and 1D). Taken together, these observations confirmed that ISG15 is highly expressed in NPC.

### High expression of ISG15 predicted more frequent recurrence in NPC

**Figure 2:**
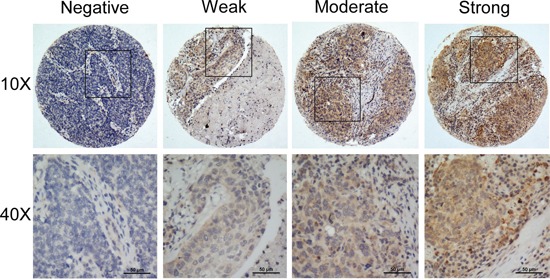
Representative IHC staining of ISG15 in NPC samples

To examine the whether the high expression of ISG15 protein is correlated with the clinical progression of NPC, IHC was performed in an independent formalin-fixed, paraffin-embedded-based (FFPE-based) tissue microarray (TMA) consisting of 209 NPC samples. Excluding the seven shedding chips, 197 of 202 (97.5%) cases showed primary cytoplasmic staining of ISG15 in cancer cells, with occasional immunoreactivity observed in the stromal lymphocytes (Figure 2). The ROC curve analysis for survival status was used to determine the cutoff score for high expression of ISG15. Tumors with scores above 5 were considered to have high ISG15 expression, leading to the greatest number of tumors classified as having or not having the clinical outcome. In our study, high ISG15 levels were detected in 63/202 (31.2%) of NPCs. The results showed no significant association between ISG15 expression and patient gender, age, histological classification, T classification, N classification, distant metastasis and clinical stages (*P*>0.05, Table 1). Whereas, ISG15 expression was correlated with tumor recurrence because high expression of ISG15 was more frequent in the NPC recurrence group than in the non-recurrence group (*P*<0.05, Table 1).

**Table 1:** Correlation between the expression of ISG15 protein and clinicopathologic features in nasopharyngeal carcinomas

	All cases	ISG15 protein		
		Low expression	High expression	*P* value^a^
Sex				0.529
Female	55	36 (65.5%)	19 (34.5%)	
Male	147	103(70.0%)	44 (30.0%)	
Age at diagnosis (years)				0.880
≤ 45	85	58 (68.2%)	27 (31.8%)	
> 45	117	81 (69.2%)	36 (30.8%)	
Histological classification (WHO)^b^				0.264
Type II	52	39 (75.0%)	13 (25.0%)	
Type III	150	100 (66.7%)	50 (33.3%)	
T classification				0.369
T1	25	20 (80.0%)	5 (20.0%)	
T2	67	47 (70.1%)	20 (29.9%)	
T3	65	45 (69.2%)	20 (30.8%)	
T4	45	27 (60.0%)	18 (40.0%)	
N classification				0.306
N0	39	29 (74.4%)	10 (25.6%)	
N1	93	59 (63.4%)	34 (36.6%)	
N2	48	37 (77.1%)	11 (22.9%)	
N3	22	14 (63.6%)	8 (36.4%)	
Clinical stage				0.343
I	9	8 (88.9%)	1 (11.1%)	
II	55	39 (70.9%)	16 (29.1%)	
III	78	55 (70.5%)	23 (29.5%)	
IV	60	37 (61.7%)	23 (38.3%)	
Distant metastasis				0.621
Yes	50	33 (66.0%)	17 (34.0%)	
No	152	106 (69.7%)	46 (30.3%)	
Locoregional recurrence				0.046
Yes	85	52 (61.2%)	33 (39.8%)	
No	117	87 (74.4%)	30 (25.6%)	

a Chi-square test;

b World Health Organization

### High expression of ISG15 indicated a shorter overall survival and disease-free survival in NPC

**Figure 3:**
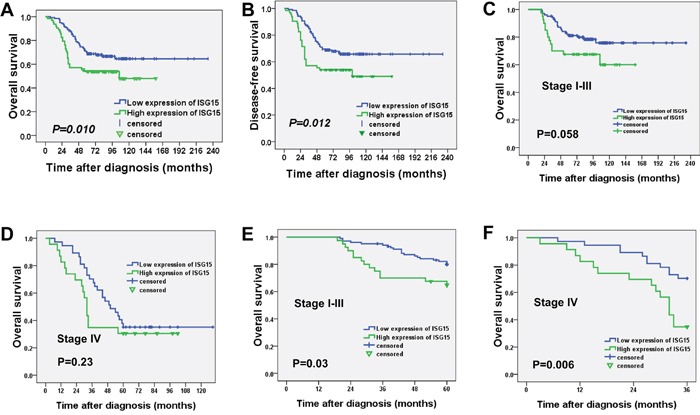
High expression of ISG15 indicated a shorter overall survival and disease-free survival in NPC patients **A.** Kaplan-Meier survival analysis of ISG15 expression for overall survival and **B.** disease-free survival (long-rank test). **C.** Kaplan-Meier survival analysis of ISG15 expression for overall survival in stage I-III and **D.** stage IV. **E.** Further analysis for 5-year overall survival in stage I-III and **F.** 3-year overall survival in stage IV.

For the 202 patients in this study, the median follow-up period was 73 months (range, 3 to 233 months), with 76 cancer-related deaths at the final clinical follow-up. The 5-year overall survival rate was 64% for the total study population. Kaplan-Meier analysis demonstrated that the patients with high ISG15 expression had significantly shorter overall survival (OS) than patients with low ISG15 expression (99.4 months vs. 166.3 months, *P*=0.010, Figure 3A). Moreover, the disease-free survival (DFS) in patients with high expression of ISG15 protein was shorter than the survival in patients with low expression of ISG15 (93.6 months vs. 166.8 months, *P*=0.012, Figure 3B). Further analysis with a Cox proportional hazards model was performed to determine whether ISG15 expression could serve as an independent prognostic predictor. A series of factors, including patients' gender, age, histological classification, T classification, N classification, clinical stages, distant metastasis, and ISG15 expression level, were included in the univariate Cox regression analysis to test their association with the OS of NPC patients. The variables most significantly associated with OS in the univariate analysis were further analyzed by multivariate analysis. The multivariate analysis model showed that ISG15 expression (HR, 1.790; 95% CI. 1.113-2.880; *P*=0.016) and clinical stage (HR, 2.386; 95% CI 1.228-4.636; *P*=0.010) were the predominant independent predictors of OS as shown in Table 2.

**Table 2:** Univariate and multivariate analysis of different prognostic variables in 202 patients with nasopharyngeal carcinoma

Variable	All cases	Univariate analysis		Multivariate analysis	
		Mean survival (months)	P value	Hazards ratio (95% CI)	P value
Sex			0.872		
Female	55	133.0			
Male	147	156.9			
Age at treatment (years)			0.272		
≤45	85	120.0			
>45	117	162.9			
Histological classification			0.104		
Type II	52	178.4			
Type III	150	121.2			
T classification			0.000	0.932 (0.670−1.296)	0.676
T1	25	155.7			
T2	67	157.2			
T3	65	178.1			
T4	45	52.8			
N classification			0.000	1.160 (0.859−1.567)	0.334
N0	39	183.2			
N1	93	162.8			
N2	48	79.9			
N3	22	61.4			
Clinical stage			0.000	2.386 (1.228−4.636)	0.010
I	9	181.0			
II	55	172.8			
III	78	172.3			
IV	60	64.4			
Distant metastasis			0.000	0.931 (0.444−1.954)	0.851
Yes	50	70.0			
No	152	171.4			
ISG15 expression			0.012	1.790 (1.113−2.880)	0.016
Low	139	166.3			
High	63	94.4			

Since the survival is usually markedly shorter in stage IV as compared with stage I-III, we performed the survival analysis concerning the ISG15 expression in stage I-III and stage IV. As shown in Figure 3C and 3D, the survival curves were separated and there were a tendency for reduced survival with high ISG15 expression in stage I-III (112.2 months vs. 188.9 months, *P*=0.058) and stage IV (48.2 months vs. 69.8 months, *P*=0.23) during a long-term follow-up. Consequently, we further analyzed the survival with 5-year and 3-year follow-up. The patients with high expression of ISG15 had a significant shorter 5-year survival in stage I-III (50.0 months vs. 56.2 months, *P*=0.03, Figure 3E) and 3-year survival in stage IV (27.2 months vs. 32.6 months, *P*=0.006, Figure 3F). Thus, ISG15 expression is still a discriminating prognostic predictors in both stage I-III and stage IV. Taken together, these results demonstrated the significant prognostic power of ISG15 protein expression as a marker of poor outcome in NPC.

### ISG15 promoted cancer stem cell-like properties in NPC cells

**Figure 4:**
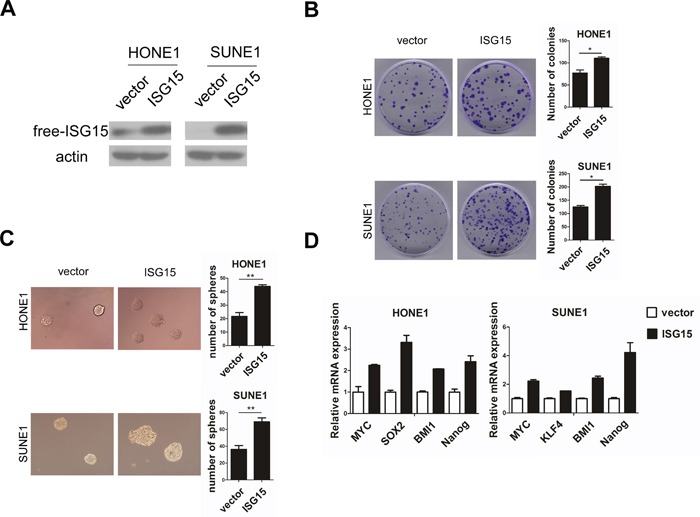
Overexpression of ISG15 promoted cancer stem cells-like property in NPC cells *in vitro* **A.** Ectopic introduction of ISG15 into HONE1 and SUNE1 cell lines. **B.** Representative micrographs and quantification of colony formation and **C.** tumorsphere formation in ISG15 overexpression or vector cells. **D.** Real-time PCR analysis of mRNA expression of pluripotency-associated markers in the indicated cells.

**Figure 5:**
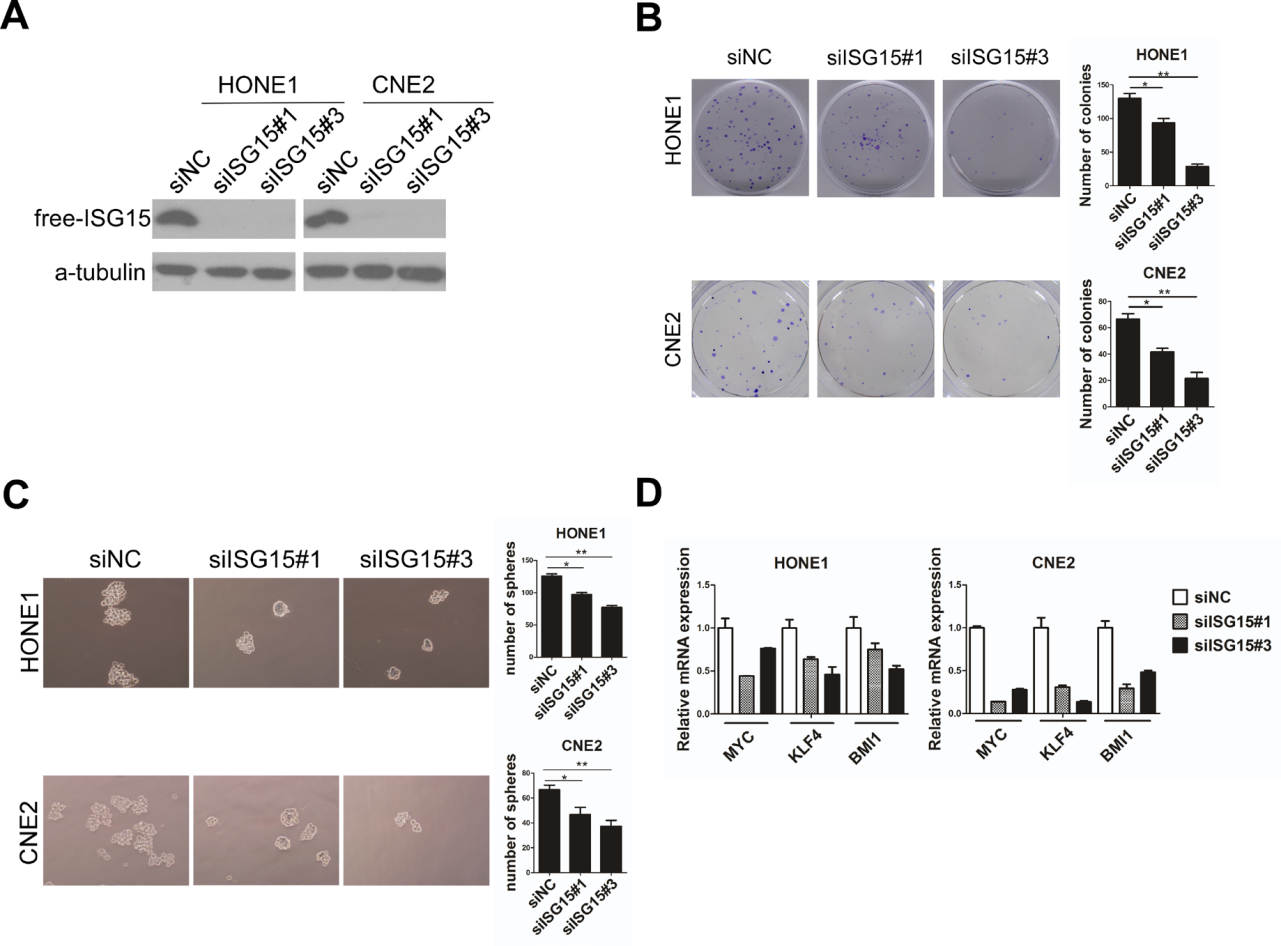
Knockdown of ISG15 attenuated cancer stem cells-like property in NPC cells *in vitro* **A.** Knockingdown ISG15 resulted in reduced ISG15 protein in HONE1 and CNE2 cell lines. **B.** Representative micrographs and quantification of colony formation and **C.** tumorsphere formation in in ISG15 knock-down and control cells. **D.** PCR analysis of mRNA expression of pluripotency-associated markers in the indicated cells.

**Figure 6:**
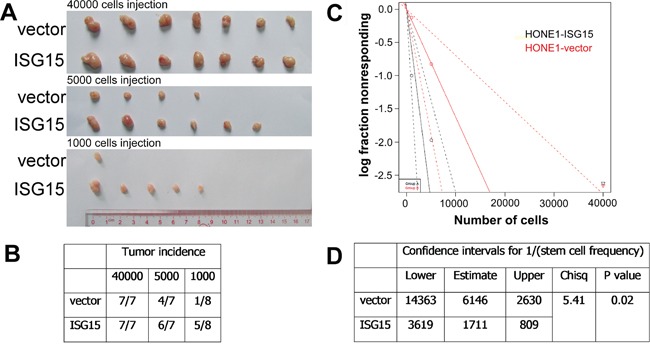
Overexpression of ISG15 increased the tumorigenicity *in vivo* **A.** and **B.** Tumor formation incidence. Mice were injected subcutaneously with the indicated numbers (4×10^4^ to 1×10^3^ cells) of vector and ISG15 overexpression cells. **C.** and **D.** Confidence intervals for 1/(stem cell frequency). The data were analyzed with ELDA (Extreme Limiting Dilution Analysis).

To determine the functions of ISG15 in NPC, we established stable HONE1 and SUNE1 cell lines with ISG15 overexpression (Figure 4A) and performed a series of *in vitro* experiments with these cells. We found that the colonies and tumorspheres formed by in ISG15 overexpression cells are significantly increased in number and size than cells expressing control vector (Figure 4B and 4C). Next, we examined the CSC markers by real time PCR analysis. Expression levels of pluripotency-associated genes, including BMI1, c-MYC, NANOG, and KLF4 increased compared with their expression in cells expressing control vector (Figure 4D). Consistently, knockdown of ISG15 expression significantly inhibited colony and tumorsphere formation in HONE1 and another NPC cell line CNE2 (Figure 5A and 5B and 5C); knockdown of ISG15 expression also reduced the pluripotency-associated gene expression levels when compared with the negative control (Figure 5D). To determine the tumorigenicity *in vivo*, we subcutaneously injected HONE1-ISG15 and vector-expressing cells into nude mice and found that ISG15 overexpression cells gave rise to more visible tumors than vector-expressing cells (Figure 6A and 6B). The estimated stem cell frequency is 1/1711 in ISG15 overexpression cells compared with 1/6146 in the vector-expressing cells (Figure 6C and 6D, *P*=0.02). Taken together, these results indicated that induction of ISG15 stimulates the stemness properties of NPC.

### ISG15 conferred resistance to chemotherapy and radiation

**Figure 7:**
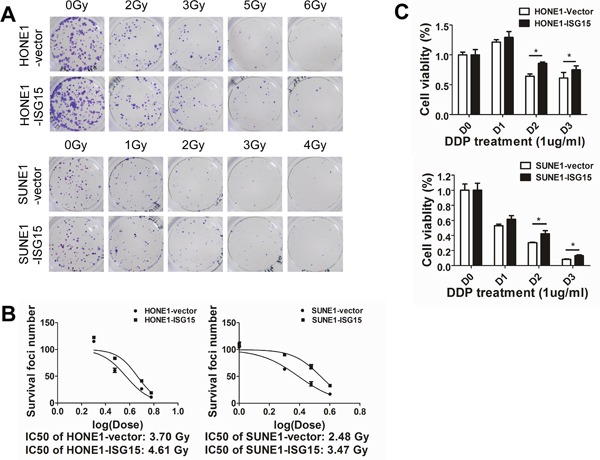
Overexpression of ISG15 enhanced NPC cell resistance to radiation and DDP treatment **A.** Clonogenic survival assays show that ISG15 overexpression increased NPC cell radioresistance compared with the vector. **B.** X-ray dose of IC50 in ISG15 overexpression cells and vector-expressing cells. **C.** Cell viability assays show that ISG15 overexpression inhibited DDP-induced NPC cell death compared with the vector.

Because ISG15 induced stem-like properties and correlated with tumor recurrence and shorter survival, we further validated whether ISG15 may confer resistance to radiation and chemotherapy. As radiotherapy is the primary treatment regimen for NPC, we detected colony formation in cells after radiation exposure. Our results revealed that overexpression of ISG15 increased radioresistance in NPC cells (Figure 7A). The X-ray dose of IC50 in overexpression cells was higher than that in the vectors (Figure 7B. HONE1-ISG15 vs. vector, 4.61 Gy vs. 3.70 Gy, *P*<0.05; SUNE1-ISG15 vs. vector, 3.47 Gy vs. 2.48 Gy, *P*<0.05). Moreover, cell viability assays were performed to evaluate DDP-induced apoptosis in ISG15 overexpression cells and vector-expressing cells. DDP-induced cell death was inhibited in ISG15 overexpression cells compared with vector-expressing cells (Figure 7C). These results suggested that ISG15 conferred resistance to chemotherapy and radiation.

## DISCUSSION

Recently, ISG15 has emerged as a promising oncoprotein elevated in various tumors [12–15]. However, it has also been reported that free ISG15 suppressed the growth of cancer cells via its immunomodulatory properties *in vivo* [18, 21]. As a result, ISG15 was considered as a “double-edged sword protein” with both antitumor and protumor functions. However, its role in NPC remains unknown. Our study was the first to show that the expression of ISG15 was higher in NPC cell lines and NPC tissues compared with the immortalized NPECs and non-cancerous nasopharyngeal tissues. Moreover, high ISG15 level was associated with more tumor recurrence and indicated a shorter overall survival and disease-free survival in NPC patients. These observations indicated that ISG15 plays a protumor role in NPC development.

ISG15 is an interferon (IFN)- α/β–inducible, ubiquitin-like intracellular protein. It can be induced by diverse microbial stimuli such as viral infection and LPS treatment [22, 23]. The nasopharynx is the uppermost region of the pharynx, which is a transitional area between the nasal cavity and the pharynx. The unique anatomic site of NPC may imply a contributing role of the microenvironment in its pathogenesis [24, 25]. Epstein–Barr virus (EBV) infection is a well-known predominant etiologic risk factor in NPC [26, 27]. An intricate interplay of EBV with host matrix and genetic alteration in infected host cells is likely to be involved in the onset and progression of NPC [25, 28]. Thus, it will be interesting to investigate whether EBV infection contributes to overexpression of ISG15 in NPC.

Intracellular ISG15 exists in both free and conjugated pools [29]. Recent studies have revealed that intracellular free ISG15 promotes tumorigenesis and metastasis of hepatocellular cancer and breast cancer [12]. In this study, we showed that ISG15 possesses a capacity for driving stem cell-like characteristics in NPC. Ectopic introduction of ISG15 resulted in elevation of colony and sphere formation, a greater expression of pluripotent gene transcripts, and *in vivo* tumorigenic activity. In contrast, knockdown of ISG15 deregulated stem cell-like features. Parallel to the present results, it was also reported that TAMs secrete ISG15, which promotes CSC phenotypes in pancreatic ductal adenocarcinoma [13]. Our observation suggests that tumor cells expressing ISG15 enhance the CSC features of cancer cells.

Cancer stem cells (CSCs), a small population of cancer cells that possess the ability of self-renewal and differentiation, are thought to be responsible for tumor initiation and progression [30, 31]. Recently, accumulating studies have demonstrated that the CSCs of NPC have played a vital role in tumor metastasis and relapse. For example, Qin et al. found that WNT5A promoted the stemness properties of NPC cells leading to metastasis and tumorigenesis [32]. It has also been reported that a stem cell-like side population in NPC was more resistant to chemotherapy and radiotherapy [33–35]. In the present study, overexpression of ISG15 in NPC cell lines enhances the resistance of radiation and DDP treatment. This is consistent with the clinically significant finding that high ISG15 expression was associated with more frequent tumor recurrence and shorter survival in NPC.

In summary, ISG15 was up-regulated in NPC and predicted frequent tumor recurrence and poor outcome in patients. *In vivo* and *in vitro* studies revealed that ISG15 promotes CSC phenotype and radiation and chemotherapy resistance in NPC. These results demonstrated the significant prognostic power of ISG15 expression. ISG15 can serve as a biomarker with poor prognostic and a therapeutic target for NPC.

## MATERIALS AND METHODS

### Patients and tissue specimens

To compare the mRNA expression levels of ISG15 among non-cancerous nasopharyngeal mucosa and primary NPCs, biopsies were obtained at the Department of Otolaryngology & Head Neck Surgery, Sun Yat-sen University Sun Yat-sen Memorial Hospital. NPC tissue microarray (TMA) analyses were performed as previously described [36, 37]. 209 cases of NPC with sufficient follow-up data were presented from the Department of Pathology, Sun Yat-sen University Cancer Center (SYSUCC).

### Cell culture

Normal primary NPECs (NPEC03 and NPEC09) [38] and immortalized NPECs (NPEC1-Bmi1, NPEC2-Bmi1) [39, 40] were cultured in Keratinocyte serum-free medium (Invitrogen, Carlsbad, CA, USA). The NPC cell lines (C666, CNE2, S18, S26, SUNE2, 5-8F, 6-10B, SUNE1, CNE1, HNE1, HK1, and HONE1) were cultured in RPMI 1640 (Invitrogen, Carlsbad, CA, USA) supplemented with 10% fetal bovine serum (FBS; Hyclone, Logan, UT, USA) in a humidified 5% CO2 incubator at 37°C.

### RNA isolation and reverse transcriptase PCR (RT-PCR) analysis

Total RNA was extracted from the tissue specimens and NPC cell lines and NPECs using the TRIzol reagent (Invitrogen, Carlsbad, CA, USA), according to the manufacturer's instructions. The reverse transcriptase kit (Promega, Madison, Wisconsin, USA) was used to synthesize the complementary DNA (cDNA) from 2 μg of the total RNA. qRT-PCR was performed using the Power SYBR Green qPCR SuperMix-UDG (Invitrogen, Carlsbad, CA, USA) to detect the mRNA level of the target genes using a LightCycler 480 II (Roche, Basel, Switzerland). β-actin was used as an internal control. The relative expression of target genes was normalized to the expression of β-actin, which yielded a2-Δct value. All reactions were performed in triplicate in three independent experiments. The sequences of the real-time PCR primers were as follows:

ISG15 sense: CGC AGA TCA CCC AGA AGA TCG

ISG15 anti-sense: TTC GTC GCA TTT GTC CAC CA

MYC sense: GCG TCC TGG GAA GGG AGA TCC GGA GC

MYC anti-sense: TTG AGG GGC ATC GTC GCG GGA GGC TG

KLF4 sense: ACG ATC GTG GCC CCG GAA AAG GAC C

KLF4 anti-sense: TGA TTG TAG TGC TTT CTG GCT GGG CTC C

NANOG sense: CAG CCC CGA TTC TTC CAC CAG TCC C

NANOG anti-sense: CGG AAG ATT CCC AGT CGG GTT CAC C

SOX2 sense: GGG AAA TGG GAG GGG TGC AAA AGA GG

SOX2 anti-sense: TTG CGT GAG TGT GGA TGG GAT TGG TG

BMI1 sense: GCT GCC AAT GGC TCT AAT GAA

BMI1 anti-sense: TGC TGG GCA TCG TAA GTA TCT T

β-actin sense: CGC GAG AAG ATG ACC CAG AT

β-actin anti-sense: GGG CAT ACC CCT CGT AGA TG

### Western blotting analysis

Cells were harvested and lysed in SDS sample buffer (62.5 mM Tris-HCl (pH 6.8), 3% sodium dodecyl sulfate (SDS), 10% glycerol, 50 mM DL-dithiothreitol (DTT), and 0.1% bromophenol blue) with protease inhibitors (Roche, Indianapolis, IN, USA). The protein concentrations were determined by the BCA method (Pierce, Thermo Fisher Scientific Inc., Rockford, IL, USA). The proteins (10 ug) were separated by SDS-PAGE and transferred to a polyvinylidene difluoride membrane. Bovine serum albumin (5%) in TBS-T (1 mol/L Tris-HCl (pH 7.5), 0.8% NaCl and 0.1%Tween 20) was used to block the membrane. Then, the membrane was incubated with anti-ISG15 (Abcam, ab131119, Cambridge, MA, USA), anti-α-tubulin (Abcam, ab126165, Cambridge, MA, USA), and anti-β-actin (Sigma-Aldrich, A5441, St.Louis, USA) antibodies at 4°C overnight. The blots were then treated with an HRP-conjugated secondary antibody (Pierce, Rockford, IL, USA).

### Immunohistochemistry

The paraffin-embedded NPC sections were deparaffinized in xylene and an alcohol gradient to rehydrate the sections. Next, the sections were treated with a Citrate Antigen Retrieval Solution (pH=8.0) in a pressure cooker for 5 min. Subsequently, 5% bovine serum albumin (BSA) in PBS (25 mM Tris, 0.8% NaCl, 2.68 mM KCl (pH 7.4)) was added to block non-specific binding, and the sections were then incubated with a mouse monoclonal anti-ISG15 antibody (1:100, Abcam, ab131119, Cambridge, MA, USA) in a moist chamber overnight at 4°C. The secondary antibodies were incubated for 45 min at 37°C on the next day. Finally, the sections were incubated in 3, 3-diaminobenzidine for 2 min and counterstained with 10% Mayer's hematoxylin before being dehydrated and mounted. As a negative control, the primary antibodies were replaced with normal rabbit serum.

Two independent pathologists who were blind to the clinical status of the patients scored the stained sections under a microscope. Semi-quantitative analysis was used to score the staining results. The intensity was scored as follows: 0, negative staining; 1, weak staining; 2, moderate staining; and 3, strong staining. According to the percentages of the positive stained areas, extent of staining was scored as follows: 1, <25% positive tumor cells; 2, 26–50%; 3, 51–75%; 4, 76–100%. The final immunoreactivity score (IRS, 0 to 12) is the product of intensity score and the extent score.

### Establishment of stable ISG15 overexpressing cell lines

Lentiviral particles were packaged and used for cell transduction according to the manufacturer's instructions (Invitrogen, San Diego, CA, USA). The HONE1 and SUNE1 cells were transfected with pLNCX2-ISG15 or pLNCX2 (Invitrogen, San Diego, CA, USA) using Lipofectamine 2000 (Invitrogen, San Diego, CA, USA) according to the manufacturer's protocol. After incubation for 24 h, the selection reagent G418 (400 μg/mL; Invitrogen, San Diego, CA, USA) was added to select stably transfected clones. Selection was continued for 14 days.

### Transfection with siRNAs against ISG15

SiRNAs targeting the mRNA of human ISG15 and the negative control (NC) (Ruibo Biotechnology Company, Guangzhou, China) were transfected into HONE1 and CNE2 cells using LipofectamineTM RNAi MAX reagent (Invitrogen, San Diego, CA, USA) according to the manufacturer's instructions. The siRNA sequences were as follows:

siISG15#1 sense: 5′-UCCUGGUGAGGAAUAACAA dTdT-3′

siISG15#1 anti-sense: 5′-dTdT AGGACCACUCCUUAUUGUU-3′

siISG15#3 sense: 5′-GCACCGUGUUCAUGAAUCU dTdT-3′

siISG15#3 anti-sense: 5′-dTdT CGUGGCACAAGUACUUAGA-3

### Colony formation assay

Cells (300 cells per well) were plated evenly in 6-well plates and cultured for 10 days. After they were fixed with methanol for 10 min, the colonies were stained with 0.5% crystal violet in 20% methanol and counted. Independent triplicate experiments were performed.

### Tumor sphere formation assays

Cells (100 cells per well) were seeded in 6-well ultra-low cluster plates for 10 days. Spheres were cultured in DMEM/F12 serum-free medium (Invitrogen, San Diego, CA, USA) supplemented with 2% B27 (Invitrogen, San Diego, CA, USA), 20 ng/ml EGF, 20 ng/ml bFGF, and 5 μg/ml insulin (Invitrogen, San Diego, CA, USA).

### Cell viability assays

The CCK8 assay was used to measure the viability of the NPC cells. ISG15-overexpressing NPC cells were seeded onto a 96-well plate at a density of 500 cells per well. After 24 h, DDP at 1 μg/ml was added into the cultures. The cells were incubated with 10 μl CCK8 for 2 h at 37°C, then cells were counted daily by reading the absorbance at 450 nm.

### Tumorigenesis *in vivo*

Female BALB/c (nu/nu) nude mice at 4-6 weeks of age were purchased from the Guangdong Medical Lab Animal Center Co. Ltd and maintained in microisolator cages. All animals were used in accordance with institutional guidelines, and the current experiments were approved by the Use Committee for Animal Care. For the tumorigenesis experiments, HONE1-vector and HONE1-ISG15 cells at different concentrations (1000, 5000, 40000 cells) in 100 μl DMEM were injected subcutaneously into the flank of each mouse. Three weeks after inoculation, the tumors were observed and recorded, and the mice were euthanized. The data were analyzed with ELDA (Extreme Limiting Dilution Analysis) via http://bioinf.wehi.edu.au/sofeware/elda [41].

### Statistical analysis

Student's t-test was used to compare two independent groups of data. ROC curve analysis was used to determine the cutoff value for dividing the patients into low and high ISG15 expression groups. Chi-squared tests were applied to analyze the relationship between ISG15 expression and clinicopathological status. Kaplan-Meier survival curves were plotted, and log-rank tests were performed. The significance of several variables for survival was analyzed using the Cox regression model in a multivariate analysis. A *P* value < 0.05 was considered statistically significant in all cases.
